# Effects of Institutionalization and Parental Living Status on Children’s Self-Esteem, and Externalizing and Internalizing Problems in Rwanda

**DOI:** 10.3389/fpsyt.2019.00442

**Published:** 2019-06-19

**Authors:** Epaphrodite Nsabimana, Eugène Rutembesa, Peter Wilhelm, Chantal Martin-Soelch

**Affiliations:** ^1^Learning and Research Unit, Hope and Homes for Children, Kigali, Rwanda; ^2^Clinical and Health Psychology Unit, Department of Psychology, University of Fribourg, Fribourg, Switzerland; ^3^Clinical Psychology Department, University of Rwanda, Kigali, Rwanda; ^4^Unit of Clinical Psychology and Psychotherapy, Department of Psychology, University of Fribourg, Fribourg, Switzerland

**Keywords:** orphan, residential child care institution, psychological adjustment, self-esteem, externalizing behavior, internalizing behavior, family, institutionalization

## Abstract

The negative effects of institutionalization on children’s wellbeing and psychological adjustment have been extensively documented. Throughout the world, particularly in developing countries, many children in residential child care institutions known as orphanages have parents, and it is not clear how this situation affects the psychological adjustment of institutionalized children. This study aimed at investigating specifically whether institutionalization impacts negatively children’s psychological adjustment defined in terms of externalizing and internalizing behavior problems and self-esteem and whether having living parents or not has an additional influence. Children were recruited in Rwanda from seven registered institutions and six primary schools. Ninety-six institutionalized children (48 orphans, who lost at least one parent, and 46 non-orphans, who had both parents living) and 84 non-institutionalized children, who lived in a family (28 orphans and 56 non-orphans) aged 9 to 16 participated. The caregivers or parents assessed externalizing and internalizing behavior problems using the Child Behavior Checklist. Children completed the Coopersmith Self-Esteem Inventory. Controlling for gender, age, and residential area, analyses of covariance revealed that institutionalized children had significantly more externalizing behavior problems than had non-institutionalized children. In addition, non-orphans had more externalizing behavior problems than had orphans, regardless of whether they lived in an institution or not. There were no group differences in internalizing behavior problems, but there was a significant main effect of the parental living status (orphans vs. non-orphans) and a significant interaction effect between parental living status and institutionalization on self-esteem. Self-esteem of non-orphans in families was significantly higher than self-esteem of the other groups. This should be considered when making the decision to place a child in an institution, especially when her or his parents are still living, and when developing supportive programs for children without adequate parental care.

## Introduction

Residential child care institutions, known as orphanages in Rwanda, rarely meet the average acceptable environmental conditions for children’s normal development (1). They often lack stable caregiving as well as open opportunities for exploration and mastery of the world (2, 3). Moreover, the removal from family and subsequent transition to an institution embodies a wide range of stress factors for the child and poses enormous challenges for the child’s psychological adjustment (4). Subsequently, compared with children raised in families, numerous studies showed that children in institutions, referred herein as institutionalized children, demonstrate poorer physical and psychosocial development outcomes such as stunting (5, 6), insecure attachment (7–9), lower intelligence quotient (IQ) (10–12), and attention and social problems (13, 14). In addition, a large body of evidence suggests that institutionalized children are consistently more vulnerable to develop behavior problems (15), psychopathological symptoms (16), and a low self-esteem (16, 17).

According to the Unicef, an “orphan” is a child below 18 years who has lost either one (single orphan) or both parents (double orphan) by any cause of death (18). In the year 2015, globally, approximately 125 million children have lost a mother or a father, and 15.1 million children have lost both parents. More than a third of all orphans live in Africa (52 million) (18). Most of these children live with the surviving parent, the grandparent(s), or other relatives (19). For example, approximately 95% of children whose parents suffer from immunodeficiency virus infection and acquired immunodeficiency syndrome (HIV/AIDS) or died of HIV/AIDS continue to live with their extended family (20). While most orphans in Africa live with their extended families (21), a number of orphans and other vulnerable children slip through the traditional family support system and end up living in residential child care institutions.

In general, residential child care system is understood as the institutional care system for orphans (22). Children who live in such an institution are usually called orphans, despite the fact that many of them still have living parents (19). A study in Central and Eastern European and former Soviet Union countries showed that only 2% of the institutionalized children were single or double orphans (23). Globally, at least four out of five, among up to 8 million children placed in institutions, have one or both parents alive (24). In Rwanda, more than 80% of children living in institutions called orphanages are not orphans (25).

Parental loss is one of the most extreme social deprivations that a child can experience. However, the vast majority of studies on the impact of parental death have been conducted with children who currently reside with their surviving parent or another family member (26). Psychological outcomes in children who have experienced the death of a parent are heterogeneous (27). On the one hand, studies show that bereaved children more likely develop psychiatric disorders (27, 28), experience more internalizing and externalizing distress, and have a lower self-esteem than do their non-bereaved counterparts (29–31). On the other hand, studies found that death of a family member was not related to higher levels of mental health problems nor to a lower self-esteem (28–30). Thus, the impact of the loss of a parent on children’s psychosocial functioning remains unclear (32). In addition, little is known about the role of being an orphan or not in an institution.

Moreover, most of the research on the effects of institutionalization and parental loss on children was conducted in developed countries. However, results cannot easily be generalized to children in other countries, with a different economic and cultural background (32). Even within the same country, generalization of results to particular communities or cultural groups is problematic. For instance, in a study conducted in the United States of America, the effect of parental death on psychological adjustment was moderated by race. Externalizing behavior problems were significantly higher for bereaved than for non-bereaved youth in a nonminority group, but there was no difference between bereaved and non-bereaved nonminority youth (31). Similarly, other researchers have argued that institutionalization would have less or even no negative effect on children coming from disadvantaged societies, communities, or families (33–37).

Almost no scientific information about the effects of being an orphan and being institutionalized is available from Sub-Saharan Africa [for exceptions, see Refs. (38–40)]. This lack of knowledge is especially important if we take into account that the risk to become an orphan is among the highest there. Therefore, studies are necessary to fill this gap. With regard to this gap in the existing literature, we aimed to investigate the psychological adjustment of orphans and non-orphans who live either in an institution or in a family environment in a sub-Saharan country, namely, in Rwanda.

Conceptualized as an individual’s ability to effectively cope with environmental demands and associated stressors, psychological adjustment (41) has been associated with externalizing behavior problems, internalizing behavior problems, and self-esteem (32, 42). Internalizing behavior problems comprise behavioral tendencies of withdrawal, avoidance, anxiety, depression, and somatization. They refer to the tendency to express distress towards the inside (43). Externalizing behavior problems comprise aggressive and rule-breaking behavior and reflect children’s propensity to express distress outwards (44). According to Coopersmith, self-esteem is a set of basic beliefs and attitudes about the own person that is essentially shaped by the way significant people (caregivers/parents, teachers, and peers) treat a person (45).

From the literature outlined above, we derived three hypotheses: The first hypothesis (H-1) states that institutionalized children have a) more externalizing behavior problems, b) more internalizing behavior problems, and c) a lower self-esteem than have non-institutionalized children. The second hypothesis (H-2) states that children who lost at least one parent (orphans) have a) more externalizing behavior problems, b) more internalizing behavior problems, and c) a lower self-esteem than children who have both parents (non-orphans). Finally, the third hypothesis (H-3) postulates that whether children are orphans or not moderates the effect of institutionalization. The negative effect of institutionalization should be stronger for orphans than for non-orphans. Thus, in contrast to orphans and non-orphans who live in a family environment, children in institutions who are orphans have a) more externalizing behavior problems, b) more internalizing behavior problems, and c) a lower self-esteem than have children in institutions who are non-orphans. In addition, we explored whether children who lost one parent had a better psychological adjustment than had those who lost both.

To test these hypotheses, we conducted a cross-sectional study in Rwanda on children with and without parents and who lived either in institutions or in families. Rwanda is an important example for compounded adversity. The genocide against the Tutsi, severe poverty, and HIV/AIDS have had devastating consequences for the functioning of families and the larger community. They have damaged the social networks that once facilitated healthy child rearing (46, 47). Given this background, the current study sheds light on the effects of institutionalization and losing parents on the psychological adjustment of children in a poor and traumatized social environment.

## Materials and Methods

### Participants

Institutionalized children were recruited from seven institutions registered at the National Commission for Children, which were located in different geographical areas of Rwanda [urban area of Rwanda’s capital Kigali (Kicukiro and Nyarugenge districts) and rural areas of Rwanda (Kamonyi, Rubavu [rural], and Karongi [rural] districts)]. Children who lived in families were recruited in six primary schools. Schools were chosen based on their proximity to the selected institutions. The nearest school wherein the majority of the local institutionalized children were enrolled was identified as a “matching” school to that institution.

Institution managers and school directors contributed to the identification of potential children to participate in the study. Children were eligible for study recruitment if they were between 9 and 16 years old and able to communicate in Kinyarwanda. The lower age level was set to 9 years, as children at that age are able to adequately read and write. The upper level of 16 years was chosen because this is the maximum age that primary school children might have. In Rwanda, primary education and lower secondary education are known as “nine years’ basic education.” This consists of 6 years of primary education and 3 years of lower secondary education. Primary education starts at age 7 and concludes with a national examination. Delays to start school, repetition of classes following poor school results, or school dropouts are very common in Rwanda and make it likely to find 16-year-old children in primary schools.

Children suspected by their caregivers or director to have learning, mental, or physical disabilities, as well as children who did not wish to participate, were not included in the research sample. Selected children gave their informed consent to participate in the study after an information session. In addition, institution managers provided informed consent as legal guardians for institutionalized children, while parents or guardians did so for never-institutionalized children recruited from schools. Monetary transport compensation was offered to adults who had to travel in order to take part in the study.

Ethical approval for this study was obtained from the Rwanda National Ethics Committee. One hundred ninety-five children between 9 and 16 years participated in this study. However, in one institution, transfer of children into families had begun, and the institution was supposed to be closed. Therefore, this institution could not be considered to be typical, and 17 children who provided data had to be excluded from the analysis. Thus, only 178 children were eligible for data analysis: 94 of them lived in institutions, and 84 lived in families.

Of those children who lived in an institution, 34 were double orphans, who lost both parents; 14 were single orphans, who lost one parent; and 46 were non-orphans. Reasons for non-orphans to be in an institution were mostly abandonment and poverty. Roughly half of the children came to an institution when they were 3 years or younger (22 orphans and 23 non-orphans), and only a few children spent less than 4 years there (six orphans and five non-orphans). According to Rwanda’s participative community categorization of household economy, known locally as *ubudehe* categories, institutions were considered to belong to the second and third economic category (very poor and poor), which enable the satisfaction of very basic needs like food and health care (48).

Of those children who lived in a family, 16 were double orphans, nine were single orphans, and three were orphans, but the information whether one or both parents died was missing, and 56 were non-orphans. Fifty-four non-orphans lived in the family they were born in. Thirty-six lived with both parents, 15 with their mothers, three with their fathers, and two with other caregivers. According to the *ubudehe* categories, 32 of the households were very poor, 16 were poor, and eight were resource poor. Regarding orphans, four were raised by a parent, 19 by the extended family, four by an unrelated family, and one by a former institution staff. Nineteen orphans lived with two caregivers, seven with a female caregiver, and two in child-headed households. Thirteen of the households were very poor, 10 were poor, and four were resource poor (for one household, this information was missing).

The composition of gender was not significantly different between groups (Fisher’s exact test = 2.23, *p* = .514, *w* = 0.12), but the composition of residential areas was (Fisher’s exact test = 21.81, *p* < .001, *w* = 0.35). As shown in **Table 1**, more institutionalized orphans lived in urban areas, while more non-institutionalized orphans lived in rural areas. In addition, there were significant group differences in children’s age [one-way analysis of variance: *F*(3, 174) = 5.21, *p* = .002, η^2^ = .082]. Non-orphans, especially those who lived in families, were younger than orphans (see **Table 1**). Regarding time spent in the institution/family and age of placement, there was no significant difference between institutionalized non-orphans, institutionalized orphans, and non-institutionalized orphans [*F*s(1, 120) ≤ 2.95, *p*s ≥ .056, partial η^2^s ≤ .048]. However, in both variables, non-orphans in families obviously differed from those of the other groups, because 54 out of 56 children were living since their birth in their family).

**Table 1:** Sample characteristics.

	Institutionalized children				Non-institutionalized children				Total	
	Non-orphans (*n* = 46)		Orphans (*n* = 48)		Non-orphans (*n* = 56)		Orphans (*n* = 28)		(*N* = 178)	
	*n*	*%*	*n*	*%*	*n*	*%*	*N*	*%*	*N*	*%*
Girls	20	43.5	19	39.6	26	46.4	16	57.1	81	45.5
Rural residential area	19	41.3	16	33.3	28	50.0	24	85.7	87	48.9
Double orphans			34	70.8			16	64.0^a^	50	68.5^a^^b^
	*M*	*SD*	*M*	*SD*	*M*	*SD*	*M*	*SD*	*M*	*SD*
Age (years)	12.70	2.11	13.10	1.97	11.82	1.82	13.29	1.90	12.62	2.02
Years spent in institution/family	9.09	4.38	8.04	4.37	11.62	2.09	7.81	4.57	9.40	4.12
Age (years) at placement	3.61	3.67	5.06	4.22	0.20	1.35	5.78	3.96	3.27	3.99

a For three children, information was missing on whether they were single or double orphans.

b Percentage was calculated for orphans.

### Measures

The first and second authors conducted interviews with the directors of the institutions, the caregivers, and parents in the families to get background information about each child, including her or his age, whether one or both parents died, age when the child started living in the current place, the reason why the child was placed into an institution or family, and the economic background of the family.

To measure externalizing and internalizing behavior problems, we used the *Child Behavior Checklist* (*CBCL/6–18*) (49). The *Child Behavior Checklist* (CBCL) is a widely used caregiver’s report of children’s behavioral and emotional problems. The CBCL consists of 113 items (e.g., “cries a lot” and “cruelty, bullying, or meanness to others”) that capture a broad range of behavioral, physical, and emotional problems “now or within the past six months.” Parents or caregivers rate each item on a 3-point scale, ranging from 0 = “not true,” 1 = “somehow or sometimes true,” to 2 = “very true or often true.” Items are summarized to eight subscales, and several of these subscales are then combined to capture two broad-band syndrome scales. One assesses internalizing behavior problems and corresponds to the sum of the three subscales Withdrawn, Somatic Complaints, and Anxious/Depressed. The other assesses externalizing behavior problems and corresponds to the sum of the subscales Rule-Breaking Behavior and Aggressive Behavior (49).

In institutions, the director designated three caregivers closest to the child, for each participating child. The first and second authors (EN and ER) gave the designated caregivers a copy of the CBCL for each child. Then, EN and ER explained the instructions to them. Each CBCL item was read loudly by one of the three caregivers. They had to reach a consensus on their response, and the agreed response was recorded. The same procedure was applied when mothers and fathers or female and male caregivers and single parents in mono-parental families were rating the behavior of the children who were not institutionalized.

The CBCL is highly reliable and has demonstrated its validity in many studies including studies in which caregivers rated children’s behavior in residential settings (50). In our study, the Cronbach alphas for the externalizing and internalizing behavior problems scales were high (alpha = .87 and alpha = .84, respectively).

To measure self-esteem, children completed the Coopersmith Self-Esteem Inventory, school form (CSEI) (45). This self-report questionnaire is a broadly used instrument to measure global self-esteem in children and adolescents between 8 and 18 years with high reliability and proven validity (51, 52). It consists of 58 items (e.g., “I’m easy to like”) with two response options, “like me” or “not like me,” which are summarized to four self-esteem subscales and a “lie-scale” that assesses defensiveness and does not count for self-esteem. Eight items that refer to the subscale “Home-Parents” assess the quality of the relationship with parents (e.g., “My parents and I have a lot of fun together”) and children’s perception of being at home (e.g., “No one pays much attention to me at home”). These items do not fit for children in institutions and children who lost their parents and would thus produce lower scores for those children. Therefore, we replaced “parents” by “caregivers” and “home” by “in your usual environment.” Because subscales are highly correlated, we used the total self-esteem score (sum across 50 self-esteem items multiplied by 2) that ranges from 0 to 100. In our study, Cronbach’s alpha of the total score was satisfactory (alpha = .82). EN and ER individually gave a paper copy of the CSEI to every child participating in the study and instructed her or him how to complete the questionnaire.

When responses for single items were missing, we estimated the total self-esteem and externalizing and internalizing behavior problem scores by computing for each child the mean across the items with valid responses and multiplied it by the number of items that belong to that scale. However, five children did not complete the CSEI at all, and for one child, data on externalizing and internalizing behavior problems were missing.

### Translation Procedure

Both *Child Behavior Checklist* (*CBCL/6–18*) and Coopersmith Self-Esteem Inventory, school form (CSEI), were forward- and back-translated to get equivalent Kinyarwanda versions of the original English version. The first author, EN, whose mother tongue is Kinyarwanda, speaks English and is familiar with psychology terms in English, translated the instruments from English to Kinyarwanda, emphasizing conceptual rather than literal translation. A bilingual (Kinyarwanda–English) expert panel including the original translator (EN), a psychologist and an expert with experience in instrument development and translation, identified and resolved the inadequate expressions/concepts of the translation. The complete Kinyarwanda version of the questionnaires were then translated back to English by an independent translator whose mother tongue is English and who has no knowledge of the questionnaires. Discrepancies were discussed by the bilingual expert panel to get the final Kinyarwanda version.

### Data Analysis

Statistical analyses were performed with IBM SPSS Statistics 25. Because there were significant differences in age and residential area between groups (see description of the sample), we treated age and residential area as control variables. In addition, we controlled for gender, because robust gender differences in externalizing and internalizing behavior problems and self-esteem have been reported (15, 51). Externalizing and internalizing behavior problems scores were skewed to the right. To normalize the distribution, we applied a square root transformation, which also made variation within groups more homogeneous.

With the first set of analyses, we explored whether single orphans differed from double orphans by calculating a two-way (2 * 2) analysis of covariance (ANCOVA) for each dependent variable. Between-subjects factors were institutionalization (living in an institution vs. living in a family) and number of parents lost (one parent dead = single orphans vs. both parents dead = double orphans). Covariates were age, gender, and residential area, while dependent variables were self-esteem (total CSEI) and externalizing or internalizing behavior problems.

If these analyses did not show significant differences between single and double orphans, we did not distinguish these two groups anymore and computed 2 * 2 ANCOVAs to test our hypotheses. Between-subjects factors were parents’ living status (both parents alive = non-orphans vs. at least one parent dead = orphans) and institutionalization, with age, gender, and residential area as control variables.

A sensitivity analysis with the program G*Power revealed that power was sufficient (.80, α = .05) to detect moderate-to-large effects for the first set of ANCOVAs (*f* = .34; η^2^ = .103), and moderate effects for the second set of ANCOVAs (*f* = .21; η^2^ = .044) with our sample size.

## Results

### Externalizing Behavior Problems

With the first ANCOVA, single orphans were compared with double orphans. After age, gender, and residential area were controlled for [none was significant, *F*s(1, 65) ≤ 2.50, *p*s ≥ .122, partial η^2^s ≤ .036], there was no difference in square-root-transformed externalizing behavior between single and double orphans [main effect number of parents lost, *F*(1, 65) = 0.61, *p* = .806, partial η^2^ = .001], nor was there an interaction of number of parents lost with institutionalization [*F*(1, 65) = 0.18, *p* = .893, partial η^2^ < .001]. However, the main effect of institutionalization was significant and of moderate size [*F*(1, 65) = 4.59, *p* = .036, partial η^2^ = .066]. Children in institutions had more externalizing behavior problems than had children in families, which was in line with H-1a.

**Figure 1:**
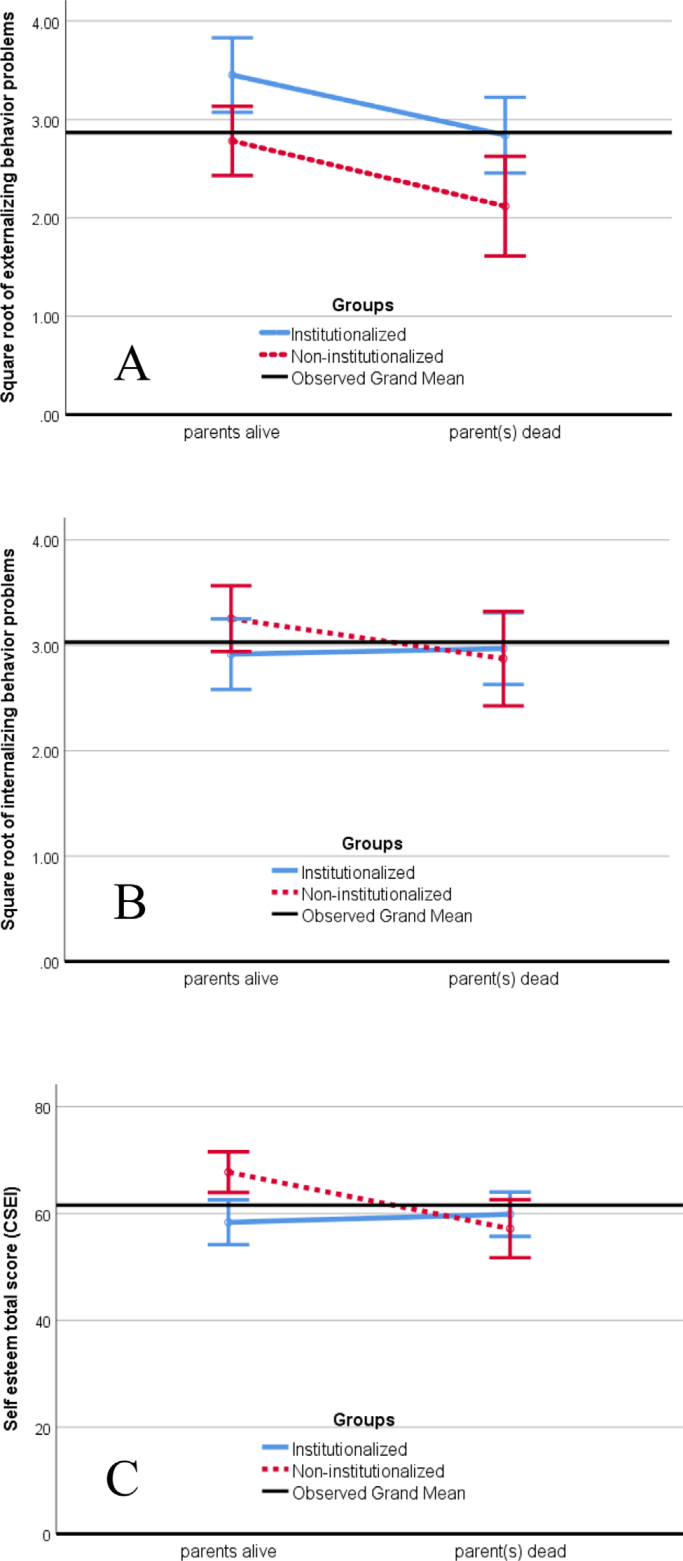
Estimated means of **(A)** square-root-transformed externalizing behavior problems, **(B)** square-root-transformed internalizing behavior problems, and **(C)** self-esteem total score for institutionalized and non-institutionalized children whose parents were alive or dead. Estimates were based on two-way analysis of covariance (ANCOVAs) with gender, age, and residential area as control variables. Error bars display 95% confidence intervals.

As single orphans were not different from double orphans, they were put into one group, and both were compared with non-orphans in the second ANCOVA. After age, gender, and residential area were controlled for [none was significant, *F*s(1, 170) ≤ 0.87, *p*s ≥ .354, partial η^2^s ≤ .005], there was no interaction effect [*F*(1, 170) = 0.16, *p* = .900, partial η^2^ < .001; rejection of H-3a], but the main effect of institutionalization was of moderate size and significant [*F*(1, 170) = 10.44, *p* = .001, partial η^2^ = .058]. As predicted, by H-1a, institutionalized children had more behavior problems than had non-institutionalized children (see **Table 2** and **Figure 1A**). There was also a moderate and significant main effect of parents’ living status [*F*(1, 170) = 9.50, *p* = .002, partial η^2^ = .053], which was, however, contrary to the prediction of H-2a. **Table 2** and **Figure 1A** show that non-orphans had even more externalizing behavior problems than had orphans.

**Table 2:** Means and standard deviations of psychological adjustment.

	Institutionalized children				Non-institutionalized children				Total	
	Non-orphans (*n* = 46)		Orphans (*n* = 47)^a^		Non-orphans (*n* = 56)		Orphans (*n* = 28)		(*N* = 177)^a^	
	*M*	*SD*	*M*	*SD*	*M*	*SD*	*M*	*SD*	*M*	*SD*
Externalizing behavior problems	13.81	10.36	10.00	9.20	8.58	5.75	6.68	6.43	10.02	8.51
Internalizing behavior problems	9.68	7.26	9.87	8.01	11.19	6.47	12.20	9.84	10.61	7.69
	(*n* = 43)^b^		(*n* = 47)^a^		(*n* = 55)^a^		(*n* = 28)		(*N* = 173)^c^	
Total self-esteem	58.64	12.44	60.16	13.48	67.99	14.61	55.73	15.67	61.56	14.61

^a^One case was missing. ^b^Three cases were missing. ^c^Five cases were missing.

### Internalizing Behavior Problems

Regarding square-root-transformed internalizing behavior problems, the first ANCOVA that compared single orphans with double orphans did not reveal any significant main or interaction effect [*F*s(1, 65) ≤ 0.79, *p*s ≥ .376, partial η^2^s ≤ .012], nor were the control variables age and gender significant [*F*s(1, 65) ≤ 0.64, *p*s ≥ .802, partial η^2^s = .001]. Only residential area had a significant effect [*F*(1, 65) = 6.22, *p* = .015, partial η^2^ = .087]. Parameter estimates revealed that children who lived in rural areas had more internalizing behavior problems than had children who lived in urban areas.

As single orphans were not different from double orphans, we compared orphans with non-orphans in the second ANCOVA. All covariates were significant [age: *F*(1, 170) = 4.44, *p* = .037, partial η^2^ = .025; gender: *F*(1, 170) = 5.93, *p* = .016, partial η^2^ = .034; residential area: *F*(1, 170) = 4.76, *p* = .030, partial η^2^ = .027]. Parameter estimates showed that older children, girls, and children who lived in rural areas had more internalizing behavior problems than had younger children, boys, and children who lived in urban areas. However, as can be seen on **Figure 1B** there was neither a significant main effect of institutionalization, nor of parents’ living status, nor a significant interaction [*F*s(1, 170) ≤ 1.40, *p*s ≥ .238, partial η^2^s ≤ .008]. These results suggest a rejection of the hypotheses for internalizing behavior problems (H-1b, H-2b, and H-3b).

### Self-Esteem

Regarding self-esteem (CSEI total score), the first ANCOVA in which single orphans were compared with double orphans did not reveal any significant main or interaction effect [*F*s(1, 65) ≤ 0.73, *p*s ≥ .396, partial η^2^s ≤ .011], nor were the control variables significant [*F*s(1, 65) ≤ 1.08, *p*s ≥ .302, partial η^2^s = .016].

As single and double orphans were not different, we compared orphans with non-orphans in the second ANCOVA. None of the covariates was significant [*F*s(1, 166) ≤ 2.59, *p*s ≥ .109, partial η^2^s ≤ .015]. Although there was no significant main effect of institutionalization on total self-esteem [*F*(1, 166) = 2.06, *p* = .153, partial η^2^ = .012], the main effect of parents’ living status [*F*(1, 166) = 4.10, *p* = .045, partial η^2^ = .024] and the interaction of parents’ living status with institutionalization were of small to medium size and became significant [*F*(1, 166) = 7.31, *p* = .008, partial η^2^ = .042]. **Table 2** and **Figure 1C** show that the difference was due to non-orphans who lived in their family. A multiple regression analysis with the three covariates and a dummy variable for each group, except for non-orphans who lived in their family (reference group), revealed that the latter group had a significantly higher total self-esteem than had children in the three other groups. After age, gender, and residential area were controlled for, self-esteem of non-orphans who lived their family was estimated to be 66.95 (*SE* = 2.44). Self-esteem of orphans in families compared with non-orphans was 10.59 (*SE* = 3.37; *t* = −3.32, *p* = .002) units lower, self-esteem of orphans in institutions was 7.88 (*SE* = 2.93; *t* = −2.70, *p* = .008) units lower, and self-esteem of non-orphans in institutions was 9.39 (*SE* = 2.88; *t* = −3.26, *p* = .001) units lower. This pattern of results rejects H-3c and suggests a modification of H-3a and H-3b.

## Discussion

The goal of the present study was to investigate psychological adjustment in orphans and non-orphans who live either in an institution or in a family environment in Rwanda. Only one result was in line with our hypotheses. We expected that psychological adjustment is worse for children who live in an institution than for those who live in a family, and we found the predicted difference for externalizing behavior problems. However, there was no such difference for internalizing behavior problems or self-esteem. Moreover, we expected that the effect of institutionalization is worse for orphans than for non-orphans, but we did not get the predicted interaction effect in the behavior problem variables. Only for self-esteem was the effect of institutionalization moderated by parents’ living status. However, the pattern of results did not confirm our prediction. Although self-esteem was low for children who lived in an institution (orphans and non-orphans), it was comparably low for orphans who lived in a family. Only non-orphans who lived in a family had a higher self-esteem.

The lower self-esteem observed in institutionalized children was in line with previous studies. Parental and subsequent social deprivation associated with institutionalization and the necessity to redefine themselves and to adapt their identity make many institutionalized children and adolescents feel insecure, lonely, and worthless, which in turn impairs their self-acceptance, self-confidence, and self-esteem (15, 53–56). However, also, non-institutionalized children who lost one or both parents need to overcome the loss and have to go through a long process of adaptation and redefinition of their self-concept, which likely decreases their self-esteem and other facets of their psychological adjustment (30, 31, 54). This reasoning might explain the lower self-esteem of never-institutionalized orphans in our study, who mostly lived with their relatives (68%) or unrelated caregivers (18%).

The finding that institutionalized children had more externalizing behavior problems than non-institutionalized children is in line with studies that reported increased externalizing behavior problems (15) and deviant behaviors for institutionalized children (57). Higher rates of externalizing behavior problems among institutionalized children might be the result of the quality of caregiver–child attachment and relations and the intensity of parenting stress within institutions which have been proven to be important factors that affect the development of children’s externalizing behaviors (58). The lack of individualized support and regimented routines, low children-to-caregiver ratio, and shift mode are frequent characteristics of institutions. Such conditions impair the bonding between children and caregivers and make it more likely that children are treated inconsistently and harshly, with little warmth and limited emotional responsiveness for their individual needs (56, 59). Moreover, children have to find their place among their peers, which is often associated with rivalry, aggression, and violence (57). In addition, institutionalized children are more likely to feel frustrated and react inadequately with deviant behavior, because they are often unable to achieve valued goals (60). Institutionalized children have been found to have elevated daily cortisol levels, which indicate ongoing stress due to a persistently activated “fight or flight” mode (61). In sum, in an institutional environment, children have difficulties in developing psychological and social skills that allow them to adequately regulate their emotions and behaviors, which in turn increases the risk to develop externalizing behavior problems (59).

In contrast to our results regarding externalizing behavior problems and our expectation (H-1b), institutionalized children did not have more internalizing behavior problems than never-institutionalized children. An explanation for this unexpected finding might be that internalizing behavior problems are more difficult to observe than externalizing behavior problems (62). In contrast to externalizing behavior problems, which are disruptive or harmful for others, internalizing behavior problems are intropunitive (63). Symptoms may fluctuate in intensity (64) and are thus more difficult to detect. In addition, internalizing behavior problems tend to be viewed as less problematic (65). A child with internalizing behavior problems is more likely to be seen as a “good” and “easy to rear” child than as a child with reportable difficulties. Consequently, the prevalence of internalizing behavior problems is lower than the prevalence of externalizing behavior problems in both orphanage and community samples when parents, caregivers, or teachers provide the information (15, 66, 67). However, contrary to the judgments of their parents, caregivers, or teachers, children report similar or even higher levels of internal compared with external behavior problems (15, 67). Thus, adults likely underestimate children’s internalizing behavior problems.

Moreover, it has been shown that caregivers’ reports are biased. Mothers report more externalizing and internalizing behavior problems than do fathers (49), and parents report more behavior problems than do teachers (65). A similar bias is likely for caregivers in institutions. Like teachers, caregivers in institutions see several children at the same time, including other children with problems. This likely increases their threshold to judge a behavior as problematic. Moreover, it is probably more difficult for caregivers in institutions to detect internalizing behavior problems than for parents or foster parents, because they work in shifts and are responsible for several children at the same time (68). In sum, we cannot exclude that such a bias might have obscured a truly existing difference in internalizing behavior problems between institutionalized and non-institutionalized children, which has been reported in other studies (15, 16).

Our second hypothesis predicted a lower psychological adjustment for orphans compared with non-orphans. It was formally confirmed by a significant main effect for self-esteem (H-2c). However, the main effect was further qualified by a stronger interaction effect, and the only children who were different and had a higher self-esteem were those who lived with their own parents. A potential explanation for these unexpected results was discussed above.

In contrast to our prediction and results reported in other studies (15, 28, 30, 31, 69), orphans did not have more internalizing behavior problems than have non-orphans (H-2b). As the demographic data show, orphans in our study have lived for many years in a new family or institution and have had time to overcome the loss of their parent(s) and adapt to the new environment. This might be a reason why withdrawal, somatic symptoms, and symptoms of depression and anxiety that are typically elevated during bereavement were not elevated in the orphans in our study. It might also explain why we did not find any difference between single and double orphans. In several of the studies that found more internalizing behavior problems for orphans compared with non-orphans, less time since the death of the parent(s) had passed (30, 31, 40) than in our study, which might explain the diverging findings.

Moreover, orphans had significantly less externalizing behavior problems, regardless of whether they lived in an institution or not. This finding was opposite to our hypothesis (H-2a). Even in institutions, orphans had less externalizing behavior problems than had non-orphans and did not exceed the level of externalizing behavior problems of non-orphans raised by their parents. Because effects were additive, children with the highest level of aggression and rule-breaking behavior were non-orphans in institutions. These findings contradict the stereotype that orphans are badly behaved and more likely to engage in defiant or socially unacceptable behaviors. Yet this is a common belief in Rwanda, which limits the willingness of the community to support orphans (70). In addition, this result is particularly concerning since children in institution who have higher rates of externalizing behavior are, once deinstitutionalized, more likely to experience family placement disruptions, which further increases their risk of externalizing behavior.

Our study has several limitations. The first limitation is its rather weak internal validity. Ideally, the answer about the effects of institutionalization would come from a randomized controlled trial. In such a trial, random assignment sends some orphans and non-orphans into institutions while others remain in a family setting (71), or orphans and non-orphans who have already been institutionalized are randomly placed into families while others remain in an institution. The Bucharest Early Intervention Project is a unique example for such a randomized controlled trial that has been underway for many years (16, 72). Such a randomized controlled trial is logistically and ethically very challenging (73).

We therefore used a better realizable naturalistic approach and conducted a quasi-experimental study. As a control group, we recruited a sample of never-institutionalized children in elementary schools, located in the direct environment of the respective institutions, who most likely share the same socioeconomic living conditions as the institutionalized children. Because we also wanted to investigate the effect of parents’ living status on institutionalization, we further distinguished orphans and non-orphans and thus had a two-factorial design with four groups.

As the description of our sample showed, the four groups were not substantially different in crucial variables, such as economic condition (poverty classification), gender composition, or single- vs. double-orphan status. Moreover, the three groups of children who did not grow up with their own parent(s) were also largely comparable regarding age of placement and time spent in the institution or family.

Nevertheless, there were some group differences. More children in institutions lived in urban areas and more children in families lived in rural areas, and children who lived with parents were younger than were children in the other groups. We therefore controlled both variables statistically by including them as covariates into the analysis. In addition, we included gender as a control variable because robust gender differences have been observed in all our indicators of psychological adjustment. Control variables were only significantly associated with internalizing behavior problems but not with externalizing behavior problems or self-esteem.

Although we did our best to exclude or control confounding variables, we cannot definitely rule out that our findings are caused by other factors than institutionalization or parents’ living status. It might be the case that children were already different before they entered an institution or family. A review of empirical studies on institutionalization revealed a number of variables that may explain the poorer adjustment among institutionalized children (46), such as impaired physical health or developmental delay that might be caused or augmented by the pre-institutionalization rearing situation. Severe social and mental health problems or alcohol and drug abuse of parent(s) more likely leads to child abandonment or neglect and finally to a separation of the child from his or her parents. This probably happens more frequently in non-orphans who are institutionalized than in orphans who are institutionalized and may lead to a greater vulnerability of the former children. This might be an alternative explanation for our finding that institutionalized non-orphans have the most externalizing behavior problems.

Moreover, research in epigenetics shows that early life stress, caused by child abuse and neglect, which might be more likely in institutionalized children, can change histone modification or DNA methylation, which then alters the way genes are expressed. This likely increases the vulnerability and risk for psychopathology later (47) and could be an additional explanation for the higher externalizing problem score of institutionalized children or the lower self-esteem scores we found. However, we cannot tell whether this was indeed the case for the institutionalized children in our study. The same is true for information about circumstances of parental death that might have had an effect on the further development of children, like deceased parent’s gender, time since death, death circumstances (74), or life events that followed parental death (27). The paucity of records, information sharing, and management in institutions in Rwanda (75) made it difficult or impossible to get reliable data about children’s background information and lived experiences before institutionalization.

A consequence of the difficulty to disentangle the various causes of problems in psychological adjustment among institutionalized children is that we do not know whether the institutional experience actually causes deficits, augments pre-existing deficits, or just maintains them (76).

The second concern refers to construct validity. We collected data with self-report and other-report instruments that were validated in Western countries. The translation–back-translation procedure to obtain a Kinyarwanda version of the CBCL and the Coopersmith Self-Esteem Inventory focused on cross-cultural and conceptual, rather than on linguistic/literal equivalence. According to our experience, externalizing and internalizing behavior problems and self-esteem are constructs that in an African cultural context may differ in nuances but will not be fundamentally different there. Therefore, we believe that the Kinyarwanda version that we have used would assess the constructs adequately, although we could not perform a validation study.

To ensure a high validity of the behavioral problem ratings, parents and caregivers who were in charge or spent the most time with the child had to find a consensus for every item. A potential problem that we cannot exclude is that caregivers and parents have a different calibration for the judgment of the severity of behavior problems. While parents seem to be more sensitive to detect and report behavior problems (77), there are several reasons (outlined above) to assume that caregivers in institutions are less sensitive to report behavior problems. Such a judgment bias would lead caregivers in institutions to underestimate the true amount of behavior problems and parents to overestimate the true amount of behavior problems. For externalizing behavior problems, the true difference between institutionalized and non-institutionalized children would then be even larger. However, such a bias might have covered a true difference between institutionalized and non-institutionalized children regarding internalizing behavior problems. We might have been able to detect such a bias if we had asked children’s teachers to complete the teachers’ form of the CBCL and had asked children to compete youth self-report form (49), in addition.

In general, our sample was rather small, especially for never-institutionalized orphans. Nevertheless, the whole sample was large enough to detect medium effects with an adequate power, when all children were included into the analyses. Therefore, it is unlikely that the nonsignificant results for internalizing behavior problems (H-1b, H-2b, and H-3b) or the missing interaction effect for externalizing behavior problems (H-1c) were due to a lack of power. Indeed, nonsignificant results were associated with effects close to zero. If we leave aside the calibration problems related to the CBCL assessment, discussed before, this would support the conclusion that these effects do not exist or are of negligible size in our population.

We collected 2014 data, at a time when a national campaign of deinstitutionalization was being conducted in Rwanda. In order to balance potential effects on our study, we targeted institutions in which deinstitutionalization programs had not yet begun and others that had formally begun. In addition, we selected institutions that were located in urban and rural areas. Non-institutionalized children should be comparable with those who were institutionalized and were therefore recruited in primary schools close to the selected institutions. Although we tried to capture the available variability between children, we only could realize convenience samples, which might have unknown biases. Therefore, we do not know how well our samples represent the population of institutionalized and non-institutionalized orphans and non-orphans in Rwanda. Nevertheless, we believe that our results reflect their situation in Rwanda and probably the situation of many children in Sub-Saharan Africa.

## Conclusion

This is the first study conducted in Rwanda that aimed to systematically investigate the effects of institutionalization and parents’ living status. Despite the above-mentioned limitations, our study provides new insights regarding the psychological adjustment of Rwandan children who live in an institution compared with those who live in families. By taking into account whether children were orphans or not, we discovered that being in an institution and not being an orphan were independently associated with higher levels of externalizing behavior problems. Thus, children who were institutionalized, although their mothers and fathers were alive, had the most externalizing behavior problems. This suggests that non-orphans are more vulnerable to the adverse effects of institutionalization than are orphans. On the other hand, orphans who lived with a family environment had the least externalizing behavior problems. Although the quasi-experimental design of our study cannot definitely rule out other interpretations, these results are in line with findings of other studies, which suggest that a family environment provides better conditions for a positive development of orphans than an institutional environment (15, 16, 78).

However, these findings were specific for externalizing behavior problems and did not generalize across the other indicators of psychological adjustment. We did not find any differences between groups regarding internalizing behavior problems. This might have been due to the fact that internalizing behavior problems are more difficult to detect than externalizing behavior problems, and that parents and caregivers may have particular biases (49, 67). Therefore, children’s and teachers’ perspective should also be taken into account when behavioral problems are assessed.

Finally, results regarding self-esteem revealed that non-orphans who lived with their parents had substantially higher values than had orphans living in families and orphans as well as non-orphans living in institutions. This suggests that not being able to live with the own parents, regardless of whether they are dead or alive, seems to impair children’s self-esteem more than living in an institution or not.

This should be considered when making the decision to place a child out of his or her family of origin. In line with the literature, our results suggest that an adequate foster family should be preferred before an institution (15, 16, 78, 79). In addition, supporting children to develop a positive self-concept and a robust self-esteem, despite difficult or adverse experiences, should be a special focus in the training of professional caregivers and in support programs for foster parents.

## Ethics Statement

All subjects gave written informed consent in accordance with the Declaration of Helsinki. The protocol was approved by Rwanda National Ethics Committee and the Internal Review Board of the Department of Psychology of the University of Fribourg/Switzerland.

## Author Contributions

EN and CM-S contributed to the conception and design of the study. ER contributed in ensuring that the protocol is in accordance to Rwanda National Ethics Committee requirements and the acquisition of data from the field. PW and EN organized the database and performed the statistical analysis. EN wrote the first draft of the manuscript. All authors contributed to the manuscript revision and have read and approved the submitted version.

## Funding

We thank the Swiss Federal Department of Foreign Affairs for granting an excellence bursary to the first author and allowing therefore this study; and finally we thank the Department of Psychology at the University of Fribourg for its financial and administrative support.

## Conflict of Interest Statement

The authors declare that the research was conducted in the absence of any commercial or financial relationships that could be construed as a potential conflict of interest. The first author EN is director of the Learning and Research Unit of Hope and Homes for Children, Kigali, Rwanda. The aim of this organization is to find families for children who still live in institutions. However, when this study was planned and conducted, EN was a doctoral student at the University of Fribourg, with a grant from the Swiss Federal Department of Foreign Affairs that guaranteed the independence of this research.

Although EN identifies with the goals of Hope and Homes for Children, the other authors hold a neutral position. Results were intensively discussed in order to eliminate or minimize any bias in the interpretation of the findings.
